# Supplementary material for: DMRforPairs: identifying Differentially Methylated Regions between unique samples using array based methylation profiles
Source: BMC Bioinformatics. 2014 May 15;15:141. doi: 10.1186/1471-2105-15-141 (PMC4046028; doi:10.1186/1471-2105-15-141)
Supplement: Additional file 2 — DMRforPairs output for the comparison of A431-MCF7 and NA17018-NA17105. Please start from the HTML files in each folder. Available via the BMC Bioinformatics website. [file 1471-2105-15-141-S2.zip › 1394847754114233_MOESM2_ESM/A431_MCF7/figures/38585.pdf]

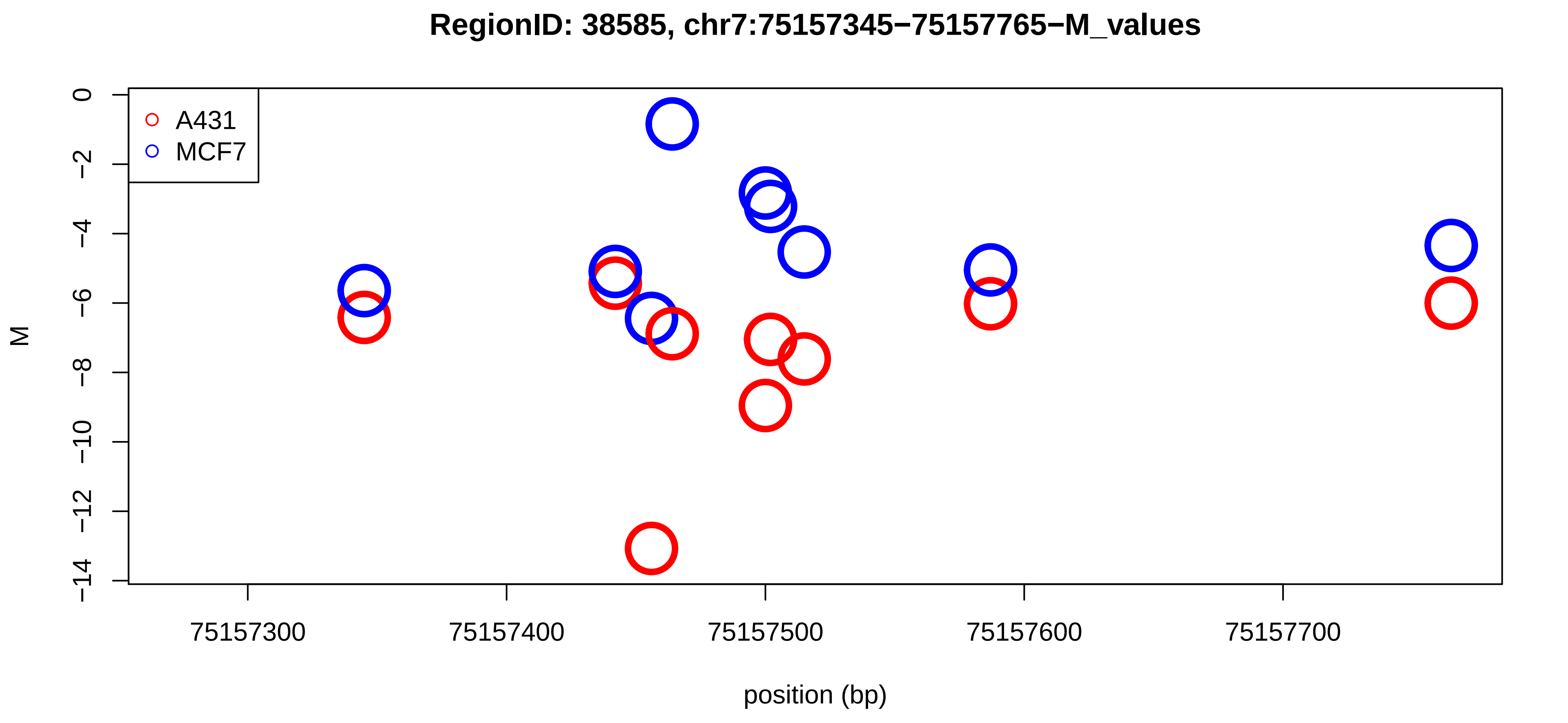

RegionID: 38585, chr7:75157345−75157765−M_values
A431
MCF7
0
−2
−4
−6
−8
−10
−12
−14
M
75157300
75157400
75157500
75157600
75157700
position (bp)


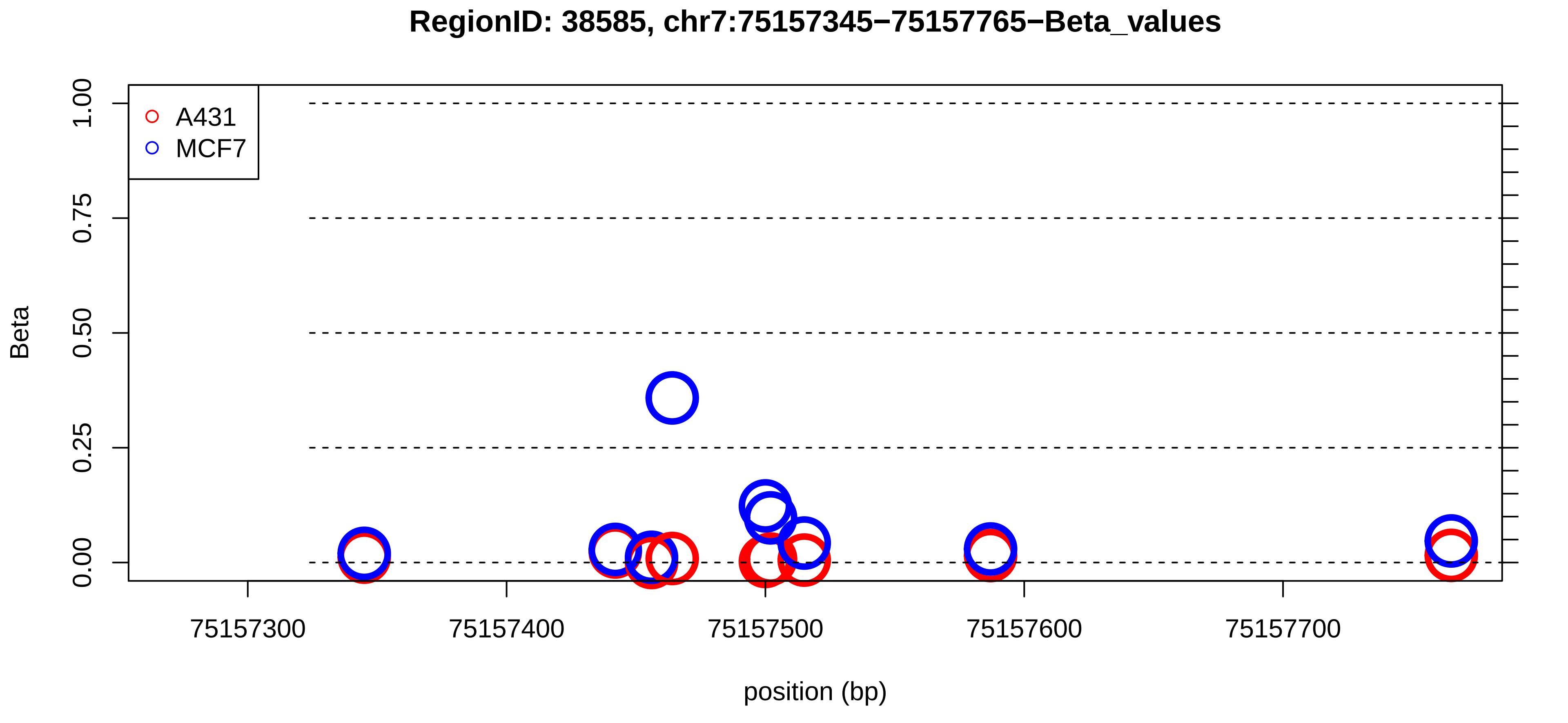

RegionID: 38585, chr7:75157345−75157765−Beta_values
A431
MCF7
1.00
0.75
0.50
0.25
0.00
Beta
75157300
75157400
75157500
75157600
75157700
position (bp)

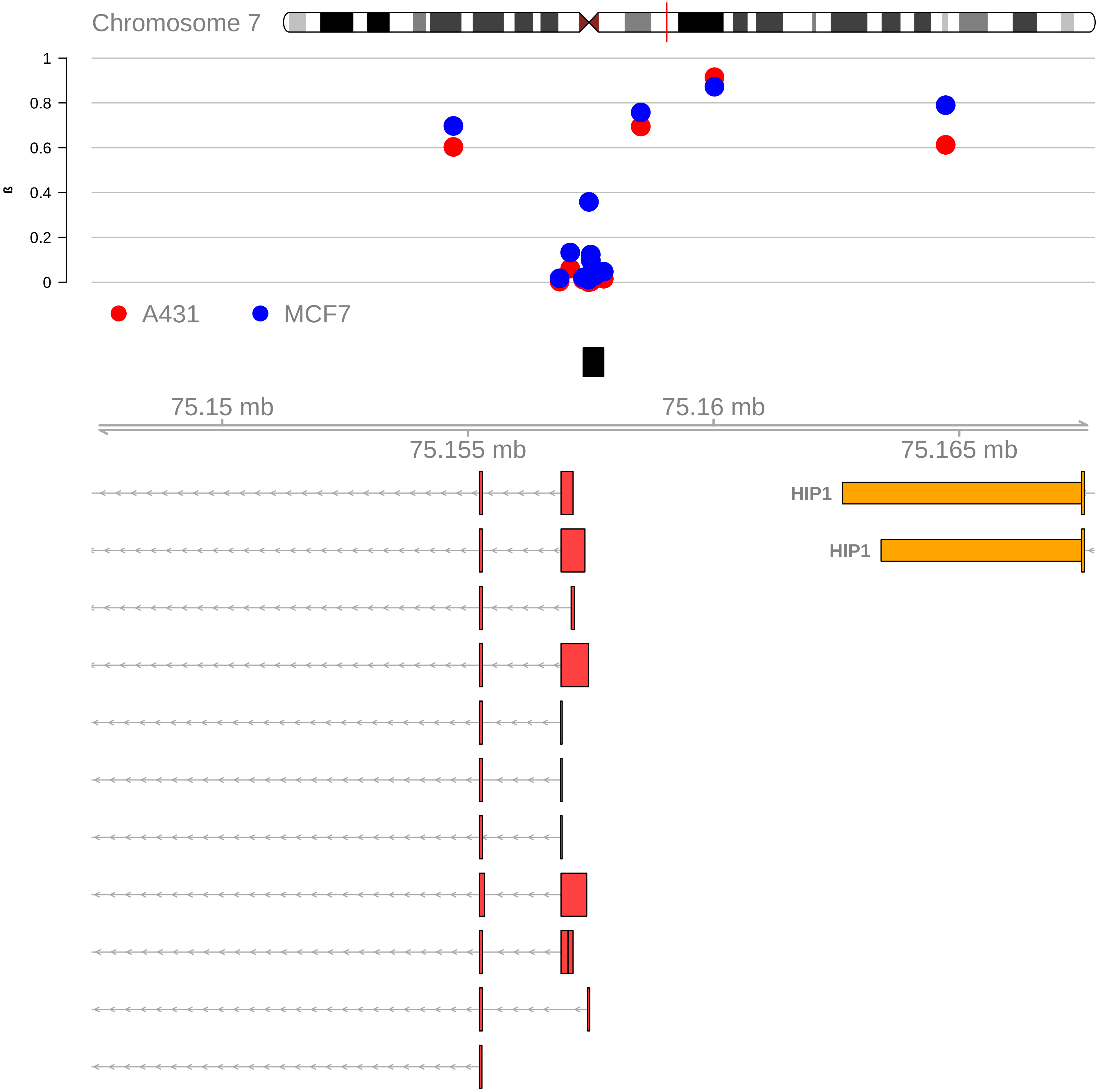

Chromosome 7
1
0.8
0.6
0.4
0.2
0
ß
A431
MCF7
75.15 mb
75.16 mb
75.155 mb
75.165 mb
HIP1
HIP1
